# Early Cost Analysis of Middle Meningeal Artery Embolization for Chronic Subdural Hematoma Treatment: A National Claims Database Analysis (MarketScan, 2007-2023)

**DOI:** 10.7759/cureus.115599

**Published:** 2026-09-01

**Authors:** Yi Zhang, Simon Levinson, Morgan Sokol, Debebe Theodros, Stephen Skirboll

**Affiliations:** 1 Neurosurgery, Stanford University, Stanford, USA; 2 Neurosurgery, Stanford Medicine, Stanford, USA

**Keywords:** administrative database, chronic subdural hematoma, cost analysis, marketscan, middle meningeal artery embolization, neurosurgery

## Abstract

Background: Middle meningeal artery (MMA) embolization has emerged as a promising, minimally invasive treatment for chronic subdural hematoma (cSDH), with randomized controlled trials demonstrating reduced recurrence compared with surgery alone. However, real-world data comparing standalone MMA embolization, surgical evacuation alone (burr hole drainage or craniotomy), and combined MMA embolization with surgical evacuation remain limited, particularly regarding comparative safety, short-term outcomes, and healthcare costs.

Methods: We conducted a retrospective analysis of MarketScan Commercial Claims and Encounters (2007-2023) identifying patients with nontraumatic SDH undergoing burr hole evacuation, craniotomy, or MMA embolization. Demographics, comorbidities, in-hospital and 30- and 90-day outcomes, costs, and reinterventions were compared across cohorts.

Results: A total of 16,291 patients were identified (7,147 burr holes, 8,757 craniotomies, 387 MMA embolizations). MMA embolization increased from 2% of cases in 2018 to 30% by 2023. MMA embolization patients had more comorbidities, including double the rate of coagulopathy (22% vs. 11-12%; p < 0.001). While index hospitalization cost for MMA embolization was the highest (median $69,383), driven by multistep interventional billing, 90-day postoperative cost was intermediate ($10,343) compared to craniotomy ($12,714) and burr hole ($6,877; p < 0.001). In-hospital mortality was lowest for MMA embolization (2%) versus burr holes (3%) and craniotomy (11%; p < 0.001). Patients undergoing combined MMA embolization and open surgery had substantially higher 90-day costs ($24,496) and stroke/transient ischemic attack (TIA) rates (40%).

Conclusions: MMA embolization has been rapidly adopted for cSDH treatment, offering favorable short-term outcomes and an intermediate cost profile to higher-risk patients. Poor outcomes with combined surgical and embolization procedures warrant further investigation. These findings support MMA embolization as practice-changing technology while highlighting the need for long-term prospective studies to optimize patient selection and cost-effectiveness.

## Introduction

Chronic subdural hematoma (cSDH) represents a significant and escalating public health challenge, with an estimated incidence of 17.3 cases per 100,000 person-years in the United States that disproportionately impacts elderly and anticoagulated patients [1-3]. Standard surgical management via burr hole drainage or craniotomy carries inherent procedural risks in frail populations and suffers from high recurrence rates requiring repeat operations [4]. Pathophysiologically, cSDH growth and recurrence are driven by fragile neovascular dural membranes primarily supplied by the middle meningeal artery (MMA) [2,5]. Endovascular MMA embolization has emerged as a promising therapy to interrupt this pathological blood supply and promote hematoma resolution [6,7].

Several high-quality randomized controlled trials (RCTs) published over the past two years have substantially strengthened the evidence supporting MMA embolization for cSDH. The landmark EMBOLISE trial demonstrated that adjunctive MMA embolization significantly reduced hematoma recurrence requiring reoperation compared with surgery alone (4.1% vs. 11.3%), with similar neurological outcomes and a low rate of serious adverse events (2.0%), supporting its adoption as a new standard adjunct to surgical care [8]. Additional multicenter RCTs, including MAGIC-MT and STEM, have independently confirmed reductions in recurrence and reintervention with a favorable safety profile [6,9-11]. Across these studies, MMA embolization was associated with lower recurrence rates without adversely affecting functional outcomes, with ongoing trials and regulatory review expected to further refine indications and patient selection. Nia et al. used the TriNetX Analytics Network to demonstrate increasing national utilization of MMA embolization as a primary treatment for cSDH, with favorable early safety and recurrence outcomes, though limited cost and health resource utilization data were reported [12].

Despite these promising clinical results, several important questions remain. Notably, there is a lack of large-scale, real-world data comparing the economic costs and health resource utilization associated with MMA embolization versus traditional surgical approaches to determine if the results of these trials are being realized in clinical practice. The heterogeneity of patient populations, procedural techniques, and definitions of recurrence in published studies further complicates direct comparisons [7,10,12-14]. Additionally, MMA embolization is often considered for patients with higher comorbidity burdens or those deemed at higher risk for surgical complications, which may influence both outcomes and costs. To address this gap, our study leverages the MarketScan commercial claims database, analyzing 16,291 patients treated for nontraumatic subdural hematoma between 2007 and 2023, to compare the economic and clinical outcomes of MMA embolization, burr hole drainage, and craniotomy for cSDH.

This study aims to characterize the early national experience with MMA embolization compared to traditional surgical approaches. We focus on procedural safety, 30- and 90-day clinical outcomes (including reintervention and major complications), and comparative healthcare costs.

## Materials and methods

Data source

Utilization data presented in this retrospective longitudinal cohort study were obtained from MarketScan Commercial Claims and Encounters, Medicare Supplemental, and Coordination of Benefits databases. These databases contain inpatient and outpatient claims detailing healthcare utilization and expenditure incurred by employees, spouses, and dependents enrolled in private health insurance plans provided by participating employers, capturing approximately 50% of the US population with employer-sponsored insurance and 40% of retirees [15]. In MarketScan, claims are organized into two distinct databases: the inpatient admissions table and the outpatient services table. Claims are categorized as inpatient if they are associated with a hospital stay involving an overnight stay (room and board), while outpatient claims encompass services provided in emergency departments or surgical centers that do not result in a formal hospital admission. All data from these databases are de-identified and therefore exempt from Institutional Review Board (IRB) approval. These data were accessed via Redivis, a research software developed in collaboration with the Stanford Center for Population Health Sciences.

Cohort definition

MarketScan databases were queried for patients with a diagnosis of nontraumatic SDH who underwent an index procedure of burr hole evacuation of subdural hematoma, craniotomy for subdural hematoma evacuation, or MMA embolization between 2007 and 2023. In clinical coding, the nontraumatic SDH designation (International Classification of Diseases, Ninth Revision (ICD-9) 432.1; ICD-10 I62.0x) is utilized to capture chronic presentations, distinguishing them from the traumatic SDH codes (ICD-10 S06.5x) reserved for acute injury [16,17]. Of note, there are no ICD codes specifically denoting chronic SDH; the nontraumatic designation, therefore, captures the majority of chronic and subacute presentations. Index hospitalization was defined as the earliest inpatient or outpatient admission date containing an ICD-9 or ICD-10 code corresponding to nontraumatic SDH, and a Current Procedural Terminology (CPT) code corresponding to one of the three index procedures. Only the first presentation of a patient undergoing a qualifying procedure was designated as the index event.

Patients were classified into three cohorts based on the index procedure: burr holes, craniotomy, or MMA embolization. Patients under 18 years of age were excluded. A full list of codes used to define the cohort and the procedures is provided in Table 1.

**Table 1:** Complete list of ICD-9/10 diagnosis codes and CPT procedure codes used to define the patient cohort and identify index procedures ICD-9/10: 9th and 10th revisions of the International Classification of Diseases; CPT: current procedural terminology; MMA: middle meningeal artery; TIA: transient ischemic attack

Variable	Codes	Type of Code
Nontraumatic subdural hematoma	432.1, I62.00, I62.01, I62.02, I62.03	ICD-9/ICD-10
Burr hole evacuation of subdural hematoma	61154	CPT
Craniotomy evacuation of subdural hematoma	61312	CPT
MMA embolization	61624	CPT
Comorbidities		
Congestive heart failure	39891, 40201, 40211, 40291, 40401, 40403, 40411, 40413, 40491, 40493, 4254, 4255, 4257, 4258, 4259, 4280, 4281, 42820, 42821, 42822, 42823, 42830, 42831, 42832, 42833, 42840, 42841, 42842, 42843, 4289, I0981, I501, I5020, I5021, I5022, I5023, I5030, I5031, I5032, I5033, I5040, I5041, I5042, I5043, I509	ICD-9/ICD-10
Cardiac arrhythmia	99601, 99604, 4260, 42610, 42611, 42612, 42613, 4267, 4269, 4270, 4271, 4272, 42731, 42732, 42741, 42742, 42760, 42761, 42769, 42781, 42789, 4279, 7850, V4500, V4501, V4502, V4509, V5331, V5332, V5339, I441, I442, I4430, I4439, I456, I459, I470, I471, I472, I479, I480, I481, I482, I483, I484, I4891, I4892, I4901, I4902, I491, I492, I493, I4940, I4949, I495, I498, I499, R000, R001, R008, T82110A, T82110D, T82110S, T82111A, T82111D, T82111S, T82118A, T82118D, T82118S, T82119A, T82119D, T82119S, T82120A, T82120D, T82120S, T82121A, T82121D, T82121S, T82128A	ICD-9/ICD-10
Valvular disease	9320, 9321, 9322, 9323, 9324, 3940, 3941, 3942, 3949, 3950, 3951, 3952, 3959, 3960, 3961, 3962, 3963, 3968, 3969, 3970, 3971, 3979, 4240, 4241, 4242, 4243, 42490, 42491, 42499, 7463, 7464, 7465, 7466, V422, V433, A5203, I050, I051, I052, I058, I059, I060, I061, I062, I068, I069, I070, I071, I072, I078, I079, I080, I081, I082, I083, I088, I089, I091, I0989, I340, I341, I342, I348, I349, I350, I351, I352, I358, I359, I360, I361, I362, I368, I369, I370, I371, I372, I378, I379	ICD-9/ICD-10
Pulmonary circulation disorders	I2601, I2602, I2609, I2690, I2692, I2699, I270, I271, I2720, I2721, I2722, I2723, I2724, I2729, I2781, I2782, I2783, I2789, I279, I280, I288, I289, 4150, 41511, 41512, 41513, 41519, 4160, 4161, 4162, 4168, 4169, 4170, 4178, 4179	ICD-9/ICD-10
Peripheral vascular disorders	930, 4373, 4400, 4401, 44020, 44021, 44022, 44023, 44024, 44029, 44030, 44031, 44032, 4404, 4408, 4409, 44100, 44101, 44102, 44103, 4411, 4412, 4413, 4414, 4415, 4416, 4417, 4419, 4431, 44321, 44322, 44323, 44324, 44329, 44381, 44382, 44389, 4439, 4471, 5571, 5579, V434, I700, I701, I70201, I70202, I70203, I70208, I70209, I70211, I70212, I70213, I70218, I70219, I70221, I70222, I70223, I70228, I70229, I70231, I70232, I70233, I70234, I70235, I70238, I70239, I70241, I70242, I70243, I70244, I70245, I70248, I70249, I7025, I70261, I70262, I70263, I70268, I70269	ICD-9/ICD-10
Hypertension uncomplicated	4010, 4011, 4019, I10,	ICD-9/ICD-10
Hypertension complicated	40200, 40201, 40210, 40211, 40290, 40291, 40300, 40301, 40310, 40311, 40390, 40391, 40400, 40401, 40402, 40403, 40410, 40411, 40412, 40413, 40490, 40491, 40492, 40493, 40501, 40509, 40511, 40519, 40591, 40599, I110, I119, I120, I129, I130, I1310, I1311, I132, I150, I151, I152, I158, I159	ICD-9/ICD-10
Paralysis	G041, G800, G801, G802, G804, G808, G809, G8100, G8101, G8102, G8103, G8104, G8110, G8111, G8112, G8113, G8114, G8190, G8191, G8192, G8193, G8194, G8220, G8221, G8222, G8250, G8251, G8252, G8253, G8254, G830, G8310, G8311, G8312, G8313, G8314, G8320, G8321, G8322, G8323, G8324, G8330, G8331, G8332, G8333, G8334, G834, G835, G8381, G8382, G8383, G8384, G8389, G839, I69031, I69032, I69033, I69034, I69039, I69041, I69042, I69043, I69044, I69049, I69051, I69052, I69053, I69054, I69059, I69061, I69062, I69063, I69064, I69065, I69069, I69131, I69132, I69133, I69134	ICD-9/ICD-10
Other neurologic disorders	E7500, E7501, E7502, E7509, E7510, E7511, E7519, E7523, E7525, E7529, E754, F842, G10, G110, G111, G112, G113, G114, G118, G119, G120, G121, G1220, G1221, G1222, G1229, G128, G129, G132, G138, G20, G214, G2401, G2402, G2409, G242, G248, G254, G255, G2581, G300, G301, G308, G309, G3101, G3109, G311, G312, G3181, G3182, G3183, G3184, G3185, G3189, G319, G3281, G35, G361, G368, G369, G370, G371, G372, G373, G374, G375, G378, G379, G40001, G40009, G40011, G40019, G40101, G40109, G40111, G40119, G40201, G40209, G40211	ICD-9/ICD-10
Chronic pulmonary disease	J40, J410, J411, J418, J42, J430, J431, J432, J438, J439, J440, J441, J449, J4520, J4521, J4522, J4530, J4531, J4532, J4540, J4541, J4542, J4550, J4551, J4552, J45901, J45902, J45909, J45990, J45991, J45998, J470, J471, J479, J60, J61, J620, J628, J630, J631, J632, J633, J634, J635, J636, J64, J65, J660, J661, J662, J668, J670, J671, J672, J673, J674, J675, J676, J677, J678, J679, J684, 4168, 4169, 490, 4910, 4911, 49120, 49121, 49122, 4918, 4919, 4920, 4928, 49300, 49301, 49302, 49310, 49311	ICD-9/ICD-10
Diabetes uncomplicated	E0800, E0801, E0810, E0811, E089, E0900, E0901, E0910, E0911, E099, E1010, E1011, E109, E1100, E1101, E119, E1300, E1301, E1310, E1311, E139, O24011, O24012, O24013, O24019, O2402, O2403, O24111, O24112, O24113, O24119, O2412, O2413, O24311, O24312, O24313, O24319, O2432, O2433, O24811, O24812, O24813, O24819, O2482, O2483, O24911, O24912, O24913, O24919, O2492, O2493, 25000, 25001, 25002, 25003, 25010, 25011, 25012, 25013, 25020, 25021, 25022, 25023, 25030, 25031, 25032, 25033	ICD-9/ICD-10
Diabetes complicated	E0821, E0822, E0829, E08311, E08319, E08321, E08329, E08331, E08339, E08341, E08349, E08351, E08359, E0836, E0839, E0840, E0841, E0842, E0843, E0844, E0849, E0851, E0852, E0859, E08610, E08618, E08620, E08621, E08622, E08628, E08630, E08638, E08641, E08649, E0865, E0869, E088, E0921, E0922, E0929, E09311, E09319, E09321, E09329, E09331, E09339, E09341, E09349, E09351, E09359, E0936, E0939, E0940, E0941, E0942, E0943, E0944, E0949, E0951, E0952, E0959, E09610, E09618, E09620, E09621, E09622, E09628, E09630, E09638, E09641, E09649, E0965, E0969, E098, E1021, E1022, E1029, E10311, E10319	ICD-9/ICD-10
Hypothyroidism	E000, E001, E002, E009, E018, E02, E030, E031, E032, E033, E038, E039, E890, 243, 2440, 2441, 2442, 2443, 2448, 2449, 2409, 2461, 2468	ICD-9/ICD-10
Renal failure	N183, N184, N185, N186, N189, N19, Z4901, Z4902, Z9115, Z940, Z992, Z4931, Z4932, 40301, 40311, 40391, 40402, 40403, 40412, 40413, 40492, 40493, 5851, 5852, 5853, 5854, 5855, 5856, 5859, 586, 5880, V560, V561, V562, V5631, V5632, V568, V4511, V4512, V420,	ICD-9/ICD-10
Liver disease	B180, B181, B182, I8500, I8501, I8510, I8511, K700, K702, K7030, K7031, K7040, K7041, K709, K7210, K7211, K7290, K7291, K730, K731, K732, K738, K739, K740, K741, K742, K743, K744, K745, K7460, K7469, K754, K7581, K760, K766, K7689, K769, Z944, 7022, 7023, 7032, 7033, 7044, 7054, 4560, 4561, 45620, 45621, 570, 5710, 5711, 5712, 5713, 57140, 57141, 57142, 57149, 5715, 5716, 5718, 5719, 5722, 5723, 5724, 5728, 5733, 5734, 5738, 5739, 706, 709, V427,	ICD-9/ICD-10
Peptic ulcer disease excluding bleeding	K254, K255, K256, K257, K259, K264, K265, K266, K267, K269, K274, K275, K276, K277, K279, K284, K285, K286, K287, K289, 53170, 53171, 53190, 53191, 53270, 53271, 53290, 53291, 53370, 53371, 53390, 53391, 53470, 53471, 53490, 53491,	ICD-9/ICD-10
AIDS/HIV	B20, 42,	ICD-9/ICD-10
Rheumatoid arthritis/connective tissue disorders	L900, L940, L941, L943, M0500, M05011, M05012, M05019, M05021, M05022, M05029, M05031, M05032, M05039, M05041, M05042, M05049, M05051, M05052, M05059, M05061, M05062, M05069, M05071, M05072, M05079, M0509, M0510, M05111, M05112, M05119, M05121, M05122, M05129, M05131, M05132, M05139, M05141, M05142, M05149, M05151, M05152, M05159, M05161, M05162, M05169, M05171, M05172, M05179, M0519, M0520, M05211, M05212, M05219, M05221, M05222, M05229, M05231, M05232, M05239, M05241, M05242, M05249, M05251, M05252, M05259, M05261, M05262, M05269, M05271, M05272, M05279, M0529, M0530, M05311, M05312, M05319, M05321, M05322	ICD-9/ICD-10
Coagulopathy	D65, D66, D67, D680, D681, D682, D68311, D68312, D68318, D6832, D684, D688, D689, D691, D693, D6941, D6942, D6949, D6951, D6959, D696, D7582, O99111, O99112, O99113, O99119, O9912, O9913, 2860, 2861, 2862, 2863, 2864, 28652, 28653, 28659, 2866, 2867, 2869, 2871, 28730, 28731, 28732, 28733, 28739, 28741, 28749, 2875,	ICD-9/ICD-10
Obesity	E6601, E6609, E661, E662, E668, E669, O99210, O99211, O99212, O99213, O99214, O99215, R939, Z6830, Z6831, Z6832, Z6833, Z6834, Z6835, Z6836, Z6837, Z6838, Z6839, Z6841, Z6842, Z6843, Z6844, Z6845, Z6854, 27800, 27801, 27802, 27803	ICD-9/ICD-10
Weight loss	E40, E41, E42, E43, E440, E441, E45, E46, E640, R634, R636, 260, 261, 262, 2630, 2631, 2632, 2638, 2639, 78321, 78322, 7994,	ICD-9/ICD-10
Fluid and electrolyte disorders	E860, E861, E869, E870, E871, E872, E873, E874, E875, E876, E8770, E8771, E8779, E878, 2760, 2761, 2762, 2763, 2764, 27650, 27651, 27652, 27661, 27669, 2767, 2768, 2769, 2536,	ICD-9/ICD-10
Blood loss anemia	D500, O9081, O99011, O99012, O99013, O99019, O9902, O9903, 2800	ICD-9/ICD-10
Deficiency anemia	D501, D508, D509, D510, D511, D512, D513, D518, D519, D520, D521, D528, D529, D530, D531, D532, D538, D539, D630, D631, D638, D649, 2801, 2808, 2809, 2810, 2811, 2812, 2813, 2814, 2818, 2819,	ICD-9/ICD-10
Alcohol abuse	F1010, F10120, F10121, F10129, F1014, F10150, F10151, F10159, F10180, F10181, F10182, F10188, F1019, F1020, F1021, F10220, F10221, F10229, F10230, F10231, F10232, F10239, F1024, F10250, F10251, F10259, F1026, F1027, F10280, F10281, F10282, F10288, F1029, F10921, F1094, F10950, F10951, F10959, F1096, F1097, F10980, F10981, F10982, F10988, F1099, 2652, 2911, 2912, 2913, 2915, 29181, 29182, 29189, 2919, 30300, 30301, 30302, 30303, 30390, 30391, 30392, 30393, 30500, 30501, 30502, 30503, 3575, 4255, 53530, 53531, 5710, 5711, 5712, 5713, 9800, 9801, 9802, 9803, 9808	ICD-9/ICD-10
Drug abuse	F1110, F11120, F11121, F11122, F11129, F1114, F11150, F11151, F11159, F11181, F11182, F11188, F1119, F1120, F1121, F11220, F11221, F11222, F11229, F1123, F1124, F11250, F11251, F11259, F11281, F11282, F11288, F1129, F1210, F12120, F12121, F12122, F12129, F12150, F12151, F12159, F12180, F12188, F1219, F1220, F1221, F12220, F12221, F12222, F12229, F12250, F12251, F12259, F12280, F12288, F1229, F1310, F13120, F13121, F13129, F1314, F13150, F13151, F13159, F13180, F13181, F13182, F13188, F1319, F1320, F1321, F13220, F13221, F13229, F13230, F13231, F13232, F13239, F1324, F13250, F13251, F13259, F1326, F1327	ICD-9/ICD-10
Psychoses	F200, F201, F202, F203, F205, F2081, F2089, F209, F22, F23, F24, F250, F251, F258, F259, F28, F29, F3010, F3011, F3012, F3013, F302, F303, F304, F308, F309, F310, F3110, F3111, F3112, F3113, F312, F3130, F3131, F3132, F314, F315, F3160, F3161, F3162, F3163, F3164, F3170, F3171, F3172, F3173, F3174, F3175, F3176, F3177, F3178, F3181, F3189, F319, F324, F325, F3340, F3341, F3342, F348, F349, F39, F4489, F843, 2970, 2971, 2972, 2973, 2978, 2979, 2980, 2981, 2982, 2983, 2984, 2988, 2989, 29604, 29614	ICD-9/ICD-10
Depression	F320, F321, F322, F323, F328, F3281, F3289, F329, F330, F331, F332, F333, F338, F339, F341, F4321, 29620, 29621, 29622, 29623, 29624, 29625, 29626, 29630, 29631, 29632, 29633, 29634, 29635, 29636, 29650, 29651, 29652, 29653, 29654, 29655, 29656, 3004, 3090, 3091, 30921, 30922, 30923, 30924, 30928, 30929, 3093, 3094, 30981, 30982, 30983, 30989, 3099, 311,	ICD-9/ICD-10
Complications		
Neurologic complication	9970, 430, 431, 432, 433, 434, 435, 456, 4382, 4383, 4384, 4385,	ICD-9/ICD-10
Iatrogenic neurologic complication	9970	ICD-9/ICD-10
Acute but ill-defined cerebrovascular disease	436	ICD-9/ICD-10
Hemiplegia	4382	ICD-9/ICD-10
Monoplegia, lower limb	4384	ICD-9/ICD-10
Monoplegia, upper limb	4383	ICD-9/ICD-10
Occlusion of cerebral arteries	434	ICD-9/ICD-10
Occlusion of precerebral arteries	433	ICD-9/ICD-10
Quadriplegia	4385	ICD-9/ICD-10
Stroke or TIA	43301, 43311, 43321, 43331, 43381, 43391, 43401, 43411, 43491, 4350, 4351, 4352, 4353, 4358, 4359, 436, G450, G451, G452, G453, G454, G458, G459, G460, G461, G462, G463, G464, G465, G466, G467, G468, H3400, H3401, H3402, H3403, H3410, H3411, H3412, H3413, H34211, H34212, H34213, H34219, H34231, H34232, H34233, H34239, I6300, I63011, I63012, I63013, I63019, I6302, I63031, I63032, I63033, I63039, I6309, I6310, I63111, I63112, I63113, I63119, I6312, I63131, I63132, I63133, I63139, I6319, I6320, I63211, I63212, I63213, I63219, I6322, I63231, I63232, I63233, I63239, I6329, I6330, I63311, I63312, I63313, I63319, I63321, I63322, I63323, I63329, I63331, I63332, I63333, I63339, I63341, I63342, I63343, I63349, I6339, I6340, I63411, I63412, I63413, I63419, I63421, I63422, I63423, I63429, I63431, I63432, I63433, I63439, I63441, I63442, I63443, I63449, I6349, I6350, I63511, I63512, I63513, I63519, I63521, I63522, I63523, I63529, I63531, I63532, I63533, I63539, I63541, I63542, I63543, I63549, I6359, I636, I638, I6381, I6389, I639, I6501, I6502, I6503, I6509, I651, I6521, I6522, I6523, I6529, I658, I659, I6601, I6602, I6603, I6609, I6611, I6612, I6613, I6619, I6621, I6622, I6623, I6629, I663, I668, I669, I6930, I6931, I69310, I69311, I69312, I69313, I69314, I69315, I69318, I69319, I69320, I69321, I69322, I69323, I69328, I69331, I69332, I69333, I69334, I69339, I69341, I69342, I69343, I69344, I69349, I69351, I69352, I69353, I69354, I69359, I69361, I69362, I69363, I69364, I69365, I69369, I69390, I69391, I69392, I69393, I69398, I6980, I6981, I69810, I69811, I69812, I69813, I69814, I69815, I69818, I69819, I69820, I69821, I69822, I69823, I69828, I69831, I69832, I69833, I69834, I69839, I69841, I69842, I69843, I69844, I69849, I69851, I69852, I69853, I69854, I69859, I69861, I69862, I69863, I69864, I69865, I69869, I69890, I69891, I69892, I69893, I69898, I6990, I6991, I69910, I69911, I69912, I69913, I69914, I69915, I69918, I69919, I69920, I69921, I69922, I69923, I69928, I69931, I69932, I69933, I69934, I69939, I69941, I69942, I69943, I69944, I69949, I69951, I69952, I69953, I69954, I69959, I69961, I69962, I69963, I69964, I69965, I69969, I69990, I69991, I69992, I69993, I69998, P91821, P91822, P91823, P91829	ICD-9/ICD-10
Infection	9985, 99859, T814, T8141XA,T8142XA	ICD-9/ICD-10
Myocardial infarction	410, 41001, 41002, 4101, 41011, 41012, 4102, 41021, 41022, 4103, 41031, 41032, 4104, 41041, 41042, 4105, 41051, 41052, 4106, 41061, 41062, 4107, 41071, 41072, 4108, 41081, 41082, 4109, 41091, 41092, I21, I210, I211, I212, I213, I214, I219, I22, I220, I221, I228, I229, I23, I230, I231, I232, I233, I234, I235, I236, I238	ICD-9/ICD-10
Pulmonary embolism	4151, 41511, 41513, 41519, I26, I260, I269, I2699	ICD-9/ICD-10
Seizure	345, 3450, 34500, 34501, 3451, 34510, 3452, 3454, 34540, 34541, 3455, 34550, 345501, 3456, 34560, 34561, 3457, 34570, 34571, 3458, 34580, 34581, 3459, 34590, 34591, G400, G4000, G40001, G4001, G40011, G40019, G4009, G401, G4010, G40101, G40109, G4011, G40111, G40119, G40129, G402, G4020, G40211, G4030, G40301, G40309, G4031, G40311, G40319, G404, G4040, G40401, G40409, G4041, G40411, G40419, G405, G4050, G40501, G40509, G408, G4080, G40801, G40802, G40803, G40804, G4082, G40821, G40822, G40823, G40824, G409, G40901, G40909, G4091, G40911, G40919, G40A, G40A0, G40A01, G40A09, G40A1, G40A11, G40A19	ICD-9/IC

Variables

The primary exposures of interest in this study were the index procedure type: burr hole evacuation of subdural hematoma, craniotomy for subdural hematoma evacuation, and MMA embolization. Each procedure was identified using a prespecified list of CPT and ICD-9/10 procedure codes. Outcomes of interest included the following: (1) readmission, defined as any all-cause hospitalization occurring within 30 days and 90 days of the index hospitalization; (2) time to first readmission, assessed over 30- and 90-day postdischarge periods; (3) rates of postoperative complications within 30 and 90 days, including seizure, myocardial infarction (MI), stroke or TIA, pulmonary embolism (PE), and infection; and (4) total healthcare costs, defined as total eligible charges incurred for any purpose, including patient and medical plan components, for the index hospitalization and 30-day and 90-day postdischarge periods. Costs are reported as unadjusted values, as common health price deflators exclude or weigh payments from public programs like Medicare relative to private insurers differently [18]. Because no single deflator universally captures payment adjustments across heterogeneous payer sources without introducing bias, adjustment for inflation was omitted.

Additional variables included age, sex, region of residence, insurance plan type, and a comprehensive set of Elixhauser comorbidities [19,20]. 

Statistical analysis

Categorical variables were compared across treatment cohorts using Chi-squared tests, while continuous variables were compared using analysis of variance (ANOVA) or Kruskal-Wallis tests. Regional usage rates for each procedure were calculated based on the state of index hospitalization and visualized using choropleth maps generated utilizing the ggplot2 package and open-access public domain geospatial data. Cost analyses for index hospitalization and 30-day and 90-day postoperative costs are presented as median costs and interquartile range (IQR). Of note, values where n < 10 have been redacted to maintain privacy in accordance with institutional data security policies. All statistical analyses were performed in R, version 4.4.2.

## Results

Temporal trends in procedures

A total of 16,291 patients underwent treatment for nontraumatic SDH between 2007 and 2023, including 7,147 (43.9%) burr hole evacuations, 8,757 (53.8%) craniotomies, and 387 (2.4%) MMA embolizations (Table 2). Notably, the distribution of procedure types shifted markedly beginning in 2018. The proportion of SDH cases treated with MMA embolization has increased exponentially since 2018, comprising approximately 30% of all cases by 2023 (as compared to 2.3% in 2018), while the relative use of burr holes and craniotomy declined at similar rates (Figure 1). 

**Table 2:** Patient demographics and procedure characteristics stratified by index procedure type IQR: interquartile range; EPO: Exclusive Provider Organization; HMO: Health Maintenance Organization; POS: Point of Service; PPO: Preferred Provider Organization; CDHP: Consumer-Driven Health Plan; HDHP: High Deductible Health Plan Patient demographics and procedure characteristics stratified by index procedure type. To maintain privacy, values where n < 10 have been redacted. Categorical variables are presented as N (%). Continuous variables, including index procedural cost, are reported as median IQR. Reported test statistics for categorical variables and continuous variables represent Chi-square values and F-statistics, respectively. Statistical significance was determined at p < 0.05

		Burr holes	Craniotomy	MMA embo	Test statistic	p-value
		(n = 7147)	(n = 8757)	(n = 387)		
Service setting	Outpatient	1457 (20.4%)	1488 (17.0%)	88 (22.7%)	34.36	<0.001
	Inpatient	5690 (79.6%)	7269 (83.0%)	299 (77.3%)		
Year	2007	418 (5.8%)	476 (5.4%)	<10 (<2%)	2901.8	<0.001
	2008	603 (8.4%)	721 (8.2%)	<10 (<2%)		
	2009	681 (9.5%)	749 (8.6%)	<10 (<2%)		
	2010	772 (10.8%)	825 (9.4%)	<10 (<2%)		
	2011	843 (11.8%)	910 (10.4%)	<10 (<2%)		
	2012	718 (10.0%)	934 (10.7%)	<10 (<2%)		
	2013	591 (8.3%)	739 (8.4%)	<10 (<2%)		
	2014	555 (7.8%)	797 (9.1%)	<10 (<2%)		
	2015	356 (5.0%)	513 (5.9%)	<10 (<2%)		
	2016	307 (4.3%)	445 (5.1%)	<10 (<2%)		
	2017	297 (4.2%)	423 (4.8%)	<10 (<2%)		
	2018	278 (3.9%)	341 (3.9%)	<10 (<2%)		
	2019	143 (2.0%)	182 (2.1%)	15 (3.9%)		
	2020	130 (<2%)	173 (<2%)	22 (5.7%)		
	2021	144 (2.0%)	184 (2.1%)	65 (16.8%)		
	2022	166 (2.3%)	188 (2.1%)	115 (29.7%)		
	2023	145 (2.0%)	157 (<2%)	132 (34.1%)		
Age group	18-34	142 (2.0%)	306 (3.5%)	17 (4.4%)	148.0	<0.001
	35-44	210 (2.9%)	347 (4.0%)	15 (3.9%)		
	45-54	634 (8.9%)	971 (11.1%)	54 (14.0%)		
	55-64	1624 (22.7%)	2319 (26.5%)	107 (27.6%)		
	65 and older	4537 (63.5%)	4814 (55.0%)	194 (50.1%)		
Sex	Male	4901 (68.6%)	5703 (65.1%)	244 (63.0%)	23.3	<0.001
	Female	2246 (31.4%)	3054 (34.9%)	143 (37.0%)		
Region	Northeast	1021 (14.3%)	1801 (20.6%)	96 (24.8%)	171.2	<0.001
	North Central	2258 (31.6%)	2221 (25.4%)	110 (28.4%)		
	South	2456 (34.4%)	3151 (36.0%)	123 (31.8%)		
	West	1257 (17.6%)	1453 (16.6%)	53 (13.7%)		
	Unknown	155 (2.2%)	131 (1.5%)	<10 (<2%)		
Insurance plan	Comprehensive	1951 (27.3%)	2043 (23.3%)	37 (9.6%)	144.5	<0.001
	EPO	50 (0.7%)	99 (1.1%)	<10 (<1%)		
	HMO	852 (11.9%)	1001 (11.4%)	78 (20.2%)		
	POS	350 (4.9%)	448 (5.1%)	21 (5.4%)		
	PPO	3345 (48.6%)	4332 (49.5%)	211 (54.5%)		
	POS with capitation	40 (0.6%)	47 (0.5%)	0 (0.0%)		
	CDHP	185 (2.6%)	268 (3.1%)	12 (3.1%)		
	HDHP	109 (1.5%)	180 (2.1%)	22 (5.7%)		
Index procedural payment ($)		1728.1 (1263.4-2694.4)	2697.8 (2098.7-4191.5)	1708.9 (1209.9-2955.5)	1335.1	<0.001
Had >30-day follow-up		6781 (94.9%)	7940 (90.7%)	351 (90.7%)	87.1	<0.001
Had >90-day follow-up		6104 (85.4%)	6893 (78.7%)	291 (75.2%)	109.1	<0.001
Had >365-day follow-up		4449 (62.2%)	4835 (55.2%)	145 (37.5%)	89.2	<0.001

**Figure 1:**
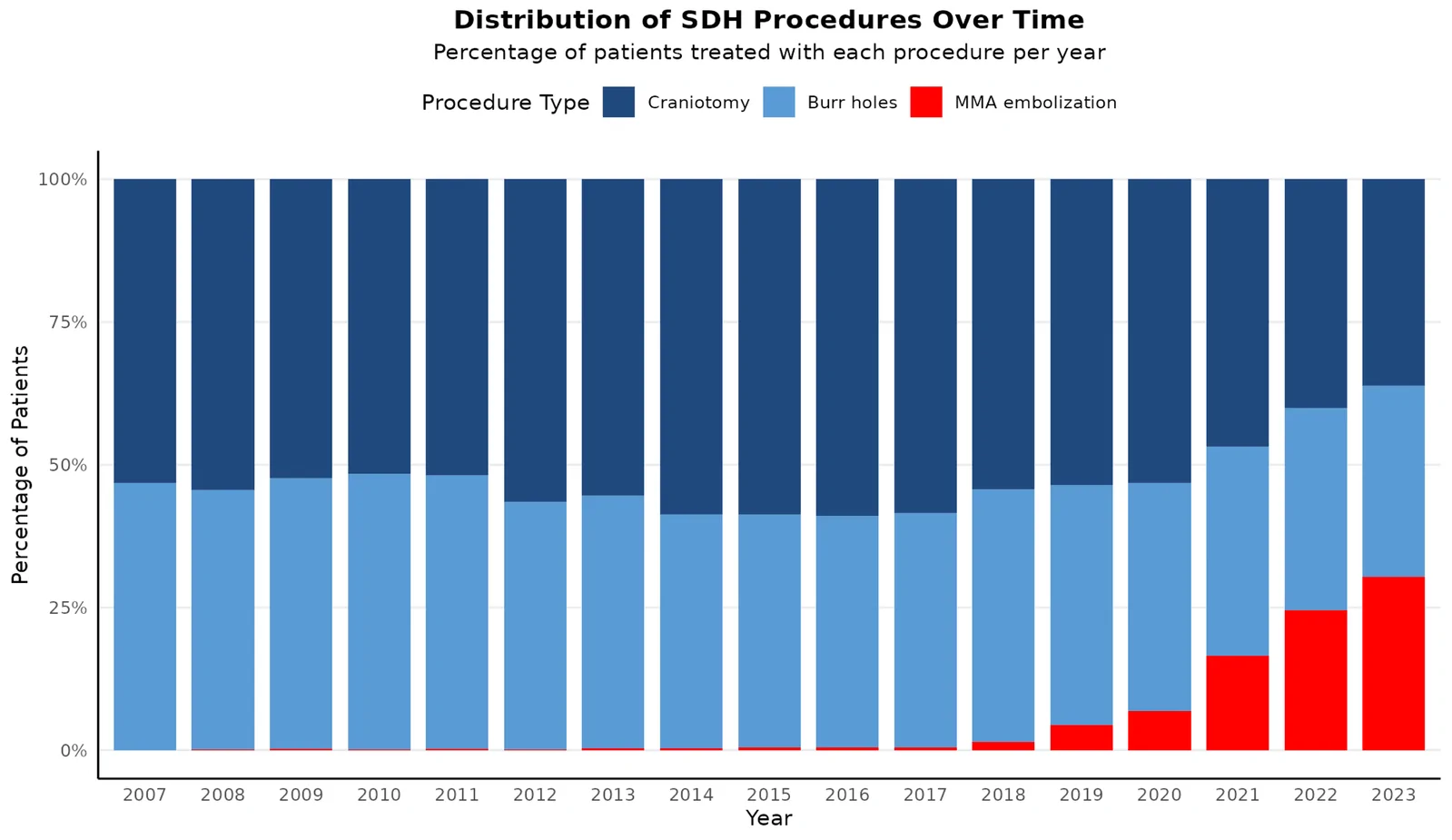
Distribution of SDH procedures over time SDH: subdural hematoma; MMA: middle meningeal artery Stacked bar chart illustrating the annual percentage of patients treated with three different procedural interventions for SDH. The colors represent the specific procedure type: craniotomy (dark blue), burr holes (light blue), and MMA embolization (red). The data demonstrates a stable proportional use of craniotomies and burr holes from 2007 to 2017. Starting in 2018, MMA embolization emerges as a treatment modality, showing steady year-over-year growth and accounting for approximately 30% of all SDH procedures by 2023

Patient and Procedure Characteristics

Patient and procedure characteristics are summarized in Table 2. The cohort was predominantly male (68.6%), although females comprised a greater proportion of MMA embolization cases (37%; p < 0.001) compared with other procedures. Patients aged 65 years and older represented the majority across all procedure types, consistent with the epidemiology of chronic SDH, with burr hole evacuations most frequently performed in this age group. Procedures were primarily performed inpatient, although outpatient cases were slightly more common among MMA embolization (22.7%) compared with burr holes (20.4%) or craniotomy (17.0%; p < 0.001).

Regional distribution varied significantly across the United States. While the South accounted for the highest absolute volume of MMA embolization cases, MMA embolization represented the highest relative proportion of the regional surgical mix in the Northeast (Figures 2A-2D). In contrast, burr hole evacuation and craniotomy procedures were more evenly distributed across the North Central, South, and Western regions.

**Figure 2:**
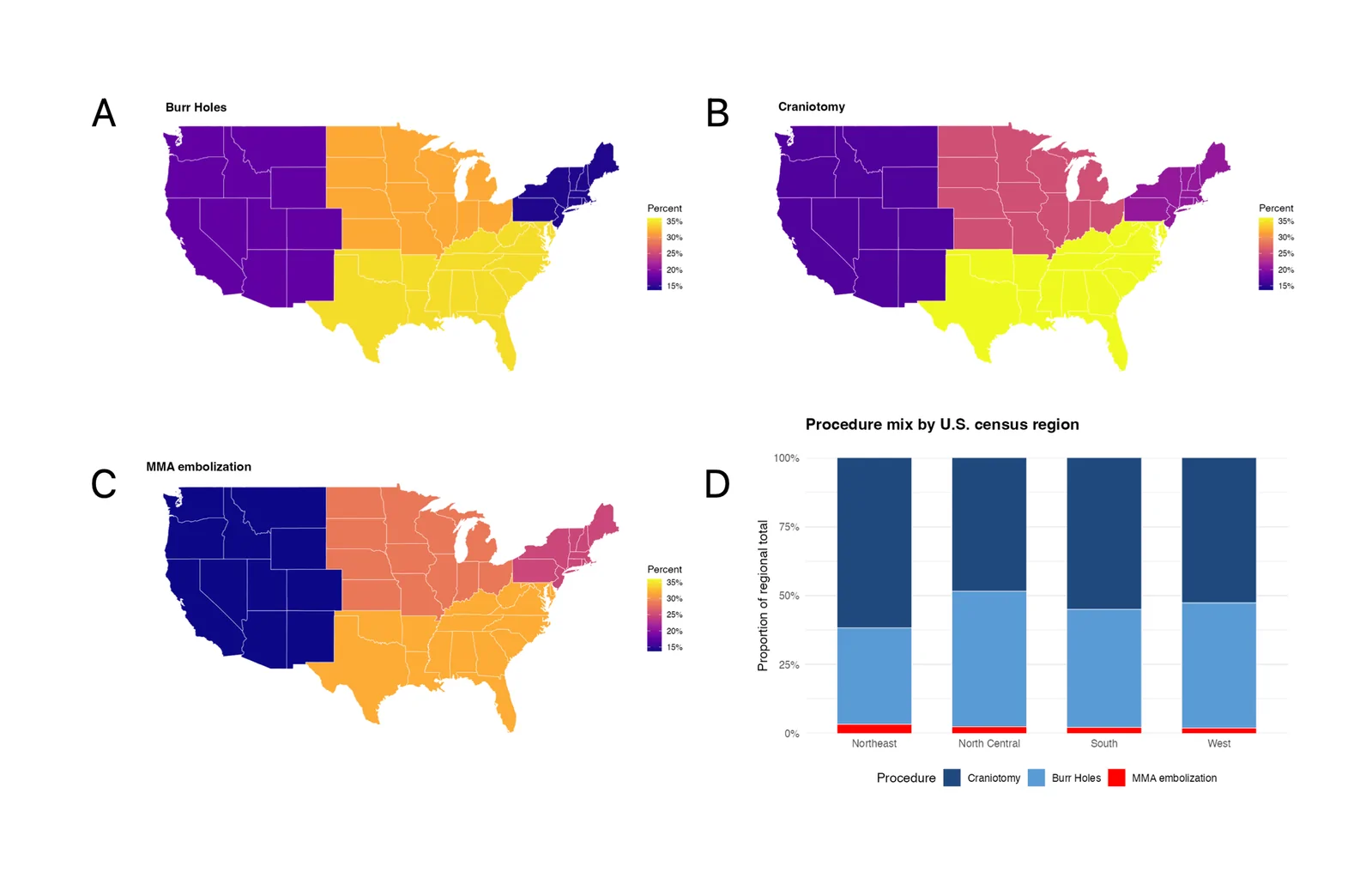
Geographic distribution and procedural mix of neurosurgical interventions by U.S. Census Region MMA: middle meningeal artery (A-C) Choropleth maps showing the regional distribution for each procedure type (burr hole evacuation, craniotomy, and MMA embolization), generated using open-source geospatial data in R. (D) Stacked bar graph illustrating procedural mix by region. MMA embolization demonstrated the highest prevalence in the Northeast, likely reflecting early adoption in academic neurointerventional centers. Cloropleth maps were generated within the open-source R statistical programming environment utilizing the ggplot2 package and open-access public domain geospatial data

Insurance type also differed significantly among procedures. Patients undergoing MMA embolization were more often enrolled in managed care plans such as Preferred Provider Organization (PPO) or Health Maintenance Organization (HMO) plans, whereas comprehensive care plans had greater representation among the burr hole and craniotomy cohorts (Table 2).

A smaller proportion of MMA embolization patients (37.5%) had ≥ 365 days of follow-up compared with burr hole (62.2%) or craniotomy (55.2%; p < 0.001) cohorts. This disparity in follow-up duration is attributable to the recent clinical adoption of MMA embolization. Consequently, to ensure statistical robustness and maximize cohort comparability, the primary focus of this study is on short-term clinical and economic outcomes at the 30-day and 90-day postprocedure intervals.

Patients who underwent MMA embolization tended to have a higher burden of baseline comorbidities, particularly for conditions associated with bleeding risk or that necessitate anticoagulation. For example, the prevalence of coagulopathy was nearly doubled in the MMA embolization group (22.2%), as opposed to 11.3% and 11.8% among burr hole and craniotomy patients, respectively (p < 0.001, Table 3). Rates of leukemia or lymphoma and alcohol use disorder were significantly higher within the MMA embolization cohort as compared to the burr hole or craniotomy cohorts. Rates of cardiac arrhythmia or valvular disorders, peripheral vascular disease, and stroke, all of which often necessitate antiplatelet agents or anticoagulation, were also significantly higher within the MMA embolization cohort as compared to the burr hole or craniotomy cohorts.

**Table 3:** Baseline Elixhauser comorbidities stratified by index procedure type To maintain privacy, values where n < 10 have been redacted. Data are presented as N (%). Reported test statistics for categorical variables represent Chi-square values. The threshold for statistical significance was set at p < 0.05

Preoperative comorbidity	Burr holes	Craniotomy	MMA embo	Test statistic	p-value
	(n = 7147)	(n = 8757)	(n = 387)		
Cancer	1005 (14.2%)	1250 (14.3%)	64 (16.5%)	1.9	0.336
Lymphoma or leukemia	149 (2.1%)	203 (2.4%)	21 (5.5%)	18.4	<0.001
Alcohol use disorder	350 (5.0%)	519 (5.9%)	26 (6.8%)	9.2	0.005
CHF	1031 (14.4%)	1240 (14.2%)	69 (17.8%)	4.1	0.177
Cardiac arrythmia or valvular disorder	2883 (40.3%)	3363 (38.4%)	189 (48.8%)	20.6	<0.001
Substance use disorder	97 (1.4%)	132 (1.6%)	7 (1.8%)	1.0	0.554
Coagulopathy	810 (11.3%)	1032 (11.8%)	85 (22.0%)	39.8	<0.001
Peripheral vascular disease	939 (13.1%)	1075 (12.3%)	82 (21.2%)	27.1	<0.001
Hypertension	4474 (62.6%)	5205 (59.4%)	264 (68.2%)	25.1	<0.001
Renal failure	720 (10.2%)	887 (10.1%)	54 (14.1%)	6.1	0.052
Liver disease	362 (5.1%)	467 (5.3%)	28 (7.3%)	3.7	0.141
Diabetes	1936 (27.1%)	2210 (25.2%)	102 (26.4%)	7.0	0.144
Stroke	1721 (24.1%)	2004 (22.9%)	124 (32.0%)	18.7	<0.001

Inpatient Costs and Outcomes

Median length of stay was comparable between MMA embolization and burr hole patients (5 days) and approximately two days longer for craniotomy patients (7 days; p < 0.001) (Table 4). Intensive care unit (ICU) utilization rates were similar across groups, with roughly 11-13.3% of patients requiring ≥ 2 days of intensive care.

**Table 4:** Inpatient outcomes and costs stratified by index procedure type, including length of stay, ICU utilization, discharge disposition, in-hospital mortality, and index admission costs ICU: intensive care unit; IQR: interquartile range; MMA: middle meningeal artery Categorical variables are reported as N (%). Continuous variables are reported as median (IQR). To maintain privacy, values where n < 10 have been redacted. Test statistics for categorical variables and continuous variables represent Chi-square values and F-statistics, respectively, except length of stay, which represents the H-statistic. The threshold for statistical significance was set at p < 0.05

	Burr holes	Craniotomy	MMA embo	Test statistics	p-value
	(n = 5690)	(n = 7269)	(n = 299)		
Length of stay (days)	5.0 (3.0-9.0)	7.0 (4.0-13.0)	5.0 (3.0-12.0)	256.7	<0.001
ICU length of stay (days)				31.8	0.002
1	5059 (88.9%)	6302 (86.7%)	261 (87.2%)		
2	536 (9.4%)	800 (11.0%)	29 (9.6%)		
3	75 (1.3%)	131 (1.8%)	<10 (<2%)		
4	11 (0.2%)	29 (0.4%)	<10 (<1%)		
5+	<10 (<1%)	<10 (<1%)	<10 (<1%)		
Cost of admission ($)	31166.9 (20244.2-49138.7)	46093.2 (29497.5-86807.3)	69382.7 (40546.4-124703.9)	658.2	<0.001
Discharge status				532.8	<0.001
Died/hospice	169 (3.0%)	777 (10.7%)	6 (2.0%)		
Home	3037 (53.4%)	2835 (39.0%)	143 (47.8%)		
Home with home health	557 (9.8%)	556 (7.6%)	41 (13.7%)		
Discharge to facility	1916 (33.7%)	3188 (43.9%)	108 (36.1%)		

Total inpatient admission cost was significantly higher for MMA embolization. Median cost for MMA embolization admissions was $69,383, more than double that of burr holes ($31,167) and approximately 50% higher than craniotomy ($46,093; p < 0.001, Table 3). While primary index procedural costs were similar, with burr hole evacuation averaging $1,728.10 and MMA embolization at $1,708.90 (and craniotomy slightly higher at $2,697.80), the total admission cost for the embolization cohort was substantially higher. 

Clinical outcomes were comparable or superior for MMA embolization relative to surgical approaches. Approximately 62% of MMA embolization patients were discharged home (with or without home health), mirroring the rate for patients who underwent burr hole evacuation (63%) and exceeding that of patients who underwent craniotomy (47%; p < 0.001). Discharge to a facility occurred in 36.1% of MMA embolization cases, 33.7% of burr hole cases, and 43.9% of craniotomy cases. Rates of in-hospital mortality or discharge to hospice were lowest for MMA embolization (2%) compared with burr holes (3%) and craniotomy (11%; p < 0.001).

30-Day Outcomes

Analysis of the 30-day postprocedure interval revealed significant differences in resource utilization, reinterventions, and complications across the cohorts. The MMA embolization cohort demonstrated a notably lower rate of all-cause 30-day readmission compared to the surgical groups. The proportion of patients with no readmissions was 75.7% in the MMA embolization cohort, which was statistically lower than the 84.2% observed in the burr hole evacuation cohort but comparable to the craniotomy cohort (76.1%; p < 0.001 overall, Table 5). Among patients who were readmitted within 30 days, median time until readmission among patients who underwent MMA embolization was 11.5 days (IQR: 5.0-17.0 days), slightly longer than patients who underwent craniotomy (10.0 days) and patients who underwent burr hole evacuation (9.0 days).

**Table 5:** The 30-day postoperative outcomes, reintervention rates, complications, and healthcare costs stratified by index procedure type TIA: transient ischemic attack; MI: myocardial infarction; PE: pulmonary embolism; IQR: interquartile range; MMA: middle meningeal artery Categorical variables are reported as N (%). Continuous variables are reported as median (IQR). To maintain privacy, values where n < 10 have been redacted. Test statistics for categorical variables and continuous variables represent Chi-square values and F-statistics, respectively, except for days until readmission, which represents the H-statistic. Statistical significance was determined at a threshold of p < 0.05

	Burr holes	Craniotomy	MMA embo	Test statistics	p-value
	(n = 6741)	(n = 7923)	(n = 345)		
Number of admissions within 30 days					
0	5673 (84.2%)	6026 (76.1%)	261 (75.7%)	151.2	<0.001
1	889 (13.2%)	1619 (20.4%)	70 (20.3%)		
2	134 (2.0%)	200 (2.5%)	10 (2.9%)		
3	43 (0.6%)	65 (0.8%)	<10 (<1%)		
4+	<10 (<1%)	13 (0.1%)	<10 (<1%)		
Days until readmission	9.0 (5.0-15.0)	10.0 (6.0-17.0)	11.5 (5.0-17.0)	54.2	<0.001
Complication coded as diagnosis for 30-day inpatient readmission					
Seizure	20 (0.3%)	45 (0.6%)	<10 (<1%)	6.3	0.551
Stroke or TIA	17 (0.3%)	32 (0.4%)	<10 (<1%)	2.5	0.929
MI	<10 (<0.5%)	<10 (<0.5%)	0 (0.0%)	0.2	0.915
PE	<10 (<0.5%)	16 (0.2%)	<10 (<1%)	11.4	0.034
Infection	12 (0.2%)	20 (0.3%)	<10 (<1%)	1.0	0.980

Within 30 days postoperatively, 6.2% of patients who underwent initial burr hole evacuation of SDH underwent another burr hole evacuation, 5.5% underwent craniotomy, and 1.2% underwent MMA embolization. In comparison, patients who underwent initial craniotomy were significantly more likely to undergo repeat craniotomy than any other procedure (11.0% compared to 1.4% for secondary burr hole evacuation, 1.0% for secondary MMA embolization). Rates of secondary burr hole evacuation, craniotomy, or MMA embolization among patients who underwent initial MMA embolization were 4.6%, 4.9%, and 4.3%, respectively.

Within the MMA embolization cohort, rates of 30-day complications such as myocardial infarction (MI), pulmonary embolism (PE), and postoperative infection were generally comparable to or lower than the surgical cohorts (Table 5). Rates of postoperative seizure were highest in the craniotomy cohort (13.1%), followed by 9.6% in the MMA embolization cohort, and 6.7% in the burr hole cohort. The rate of postoperative stroke or transient ischemic attack (TIA) was highest in the craniotomy cohort (24.9%), compared to 15.8% in the burr hole cohort and 21.4% in the MMA embolization cohort (Table 5).

MMA embolization was associated with intermediate postoperative costs, with a median 30-day all-cause postoperative cost of $5,074. In contrast, 30-day postoperative costs for craniotomy patients were higher at a median of $6,491. The burr hole evacuation cohort had the lowest 30-day costs at $3,617. These 30-day postoperative figures represent the sum of all inpatient and outpatient services incurred following the date of the index procedure; notably, this includes the costs of the remaining index hospitalization days as well as any subsequent healthcare utilization within the 30-day window. While MMA embolization carries a higher upfront cost on the day of surgery due to complex procedural billing, the subsequent 30-day financial burden is significantly higher for craniotomy patients. This difference was primarily driven by inpatient services, which were significantly higher among craniotomy patients compared to the other two cohorts (p < 0.001, Table 5). The median number of days that craniotomy patients were admitted within the 30-day postoperative window was five days, two days longer than the median for either the burr hole or MMA embolization cohorts.

Of note, patients who underwent both MMA embolization and an open surgical procedure (whether burr hole or craniotomy) had the highest healthcare costs and utilization compared to patients who underwent any single procedure alone, costing a median of $18,784 over 30 days, more than 10 times the 30-day cost of any other cohort (Table 6). They also had the highest rates of postoperative stroke or TIA (41.2%), nearly double that of stroke or TIA rates for patients who underwent craniotomy, MMA embolization, or burr hole evacuation alone (p < 0.001).

**Table 6:** The 30-day outcomes and costs for patients who underwent combined MMA embolization and open surgical procedure (burr hole or craniotomy) compared to single-modality cohorts TIA: transient ischemic attack; MI: myocardial infarction; PE: pulmonary embolism; IQR: interquartile range; MMA: middle meningeal artery Categorical variables are reported as N (%). Continuous variables are reported as median (IQR). To maintain privacy, values where n < 10 have been redacted. Test statistics for categorical variables and continuous variables represent Chi-square values and F-statistics, respectively. Statistical significance was determined at a threshold of p < 0.05

	Combined	Burr holes only	Craniotomy only	MMA embo only	Test statistics	p-value
	(n = 187)	(n = 6661)	(n = 7846)	(n = 315)		
Number of days with outpatient encounter	4.0 (2.0-8.0)	4.0 (2.0-9.0)	4.0 (2.0-9.0)	4.0 (2.0-8.0)	0.7	0.065
30-day postoperative outpatient costs ($)	1755.5 (403.0-4881.8)	1363.4 (413.2-4048.9)	1371.4 (335.8-4113.0)	1373.7 (485.5-4255.5)	1.4	0.098
Number of days with inpatient encounter	8.0 (4.0-18.0)	3.0 (1.0-6.0)	5.0 (2.0-13.0)	2.0 (0.0-7.5)	355.3	<0.001
30-day postoperative inpatient costs ($)	18783.9 (5053.6-57512.0)	526.0 (66.8-3871.0)	1805.8 (301.1-13242.3)	618.4 (0.0-9558.9)	703.9	<0.001
30-day complication rates						
Seizure	22 (11.8%)	421 (6.3%)	964 (12.3%)	27 (8.6%)	149.8	<0.001
Stroke or TIA	77 (41.2%)	988 (14.8%)	1774 (22.6%)	60 (19.0%)	197.9	<0.001
MI	<10 (<2%)	63 (0.9%)	116 (1.5%)	<10 (<2%)	8.8	0.032
PE	<10 (<2%)	130 (2.0%)	217 (2.8%)	<10 (<3%)	10.8	0.013
Infection	<10 (<2%)	47 (0.7%)	83 (1.1%)	<10 (<0.5%)	6.3	0.096

90-Day Outcomes

Over the 90-day postoperative period, 78.4% of patients who underwent burr hole evacuation remained readmission-free, significantly higher than among craniotomy or MMA embolization patients (68.0% and 65.3%, respectively; Table 7). Rates of readmission for seizure, stroke or TIA, MI, PE, or infection were not significantly different between cohorts.

**Table 7:** The 90-day readmission rates and time to readmission stratified by index procedure type TIA: transient ischemic attack; MI: myocardial infarction; PE: pulmonary embolism; IQR: interquartile range; MMA: middle meningeal artery Categorical variables are reported as N (%). Continuous variables are reported as median (IQR). To maintain privacy, values where n < 10 have been redacted. Test statistic for categorical variables and continuous variables represent Chi-square values and F-statistics, respectively, except days until readmission, which represents the H-statistic. Statistical significance was determined at a threshold of p < 0.05

	Burr holes	Craniotomy	MMA embo	Test statistics	p-value	
	(n = 6104)	(n = 6893)	(n = 291)			
Number of readmissions within 90 days				185.8	<0.001	
0	4787 (78.4%)	4687 (68.0%)	190 (65.3%)			
1	936 (15.3%)	1509 (21.9%)	69 (23.7%)			
2	242 (4.0%)	449 (6.5%)	25 (8.6%)			
3	98 (1.6%)	166 (2.4%)	<10 (<2%)			
4	30 (0.5%)	61 (0.9%)	<10 (<1%)			
5+	11 (0.2%)	21 (0.3%)	0 (0.0%)			
Days until readmission	14.0 (6.0-35.0)	13.0 (7.0-28.0)	17.0 (8.0-40.0)	33.6	0.289	
Complication coded as diagnosis for 90-day inpatient readmission						
Seizure	27 (0.4%)	61 (0.9%)	<10 (<1%)	10.0	0.260	
Stroke or TIA	<10 (<0.5%)	<10 (<0.5%)	0 (0.0%)	0.3	0.610	
MI	23 (0.4%)	41 (0.6%)	<10 (<2%)	3.3	0.964	
PE	10 (0.2%)	29 (0.4%)	0 (0.0%)	8.2	0.172	
Infection	19 (0.3%)	35 (0.5%)	<10 (<1%)	3.1	0.857	

Among patients who underwent initial burr hole evacuation, the most common secondary procedure postoperatively remained burr hole evacuation (7.9%), followed by craniotomy (6.1%) and MMA embolization (1.4%, Table 8). Among craniotomy patients, 11.7% required another craniotomy within 90 days postoperatively, 1.9% underwent secondary burr hole evacuation, and 1.0% underwent MMA embolization. Within the MMA embolization cohort, rates of secondary burr hole evacuation or craniotomy were both 6.2%, with 5.8% of patients undergoing another MMA embolization within 90 days.

**Table 8:** The 90-day reintervention rates and postoperative complications stratified by index procedure type TIA: transient ischemic attack; MI: myocardial infarction; PE: pulmonary embolism; IQR: interquartile range; MMA: middle meningeal artery Categorical variables are reported as N (%). Continuous variables are reported as median (IQR). To maintain privacy, values where n < 10 have been redacted. Test statistic for categorical variables and continuous variables represent Chi-square values and F-statistics, respectively. Threshold for statistical significance was set at p < 0.05

	Burr Holes	Craniotomy	MMA Embo	Test Statistic	p-value
	(n = 6104)	(n = 6893)	(n = 291)		
Number of days with outpatient encounter	10.0 (5.0-19.0)	12.0 (6.0-22.0)	11.0 (6.0-20.0)	37.0	<0.001
90-day outpatient costs ($)	3649.0 (1373.2-9825.9)	4687.5 [1714.6-12270.3]	4248.1 (1840.8-9037.4)	36.5	<0.001
Number of days with inpatient encounter	3.0 (1.0-7.0)	6.0 (2.0-14.0)	3.0 (0.5-11.0)	312.6	<0.001
90-day inpatient costs ($)	578.4 (78.0-6217.9)	2326.2 (346.0-20688.2)	1405.3 (16.2-23707.7)	530.5	<0.001
90-day complication rates					
Seizure	569 (9.3%)	1163 (16.9%)	32 (11.0%)	161.7	<0.001
Stroke or TIA	1237 (20.3%)	1962 (28.5%)	76 (26.1%)	139.8	<0.001
MI	81 (1.3%)	130 (1.9%)	<10 (3%)	11.8	0.036
PE	153 (2.5%)	256 (3.7%)	<10 (1%)	16.7	<0.001
Infection	65 (1.1%)	129 (1.9%)	<10 (0.5%)	17.4	<0.001
90-day reoperation rates					
Underwent subsequent burr holes	481 (7.9%)	129 (1.9%)	18 (6.2%)	261.0	<0.001
Underwent subsequent craniotomy	375 (6.1%)	805 (11.7%)	18 (6.2%)	123.8	<0.001
Underwent subsequent MMA embolization	83 (1.4%)	71 (1.0%)	17 (5.8%)	51.4	<0.001

Stroke or TIA remained the most common postoperative complication at 90 days, with rates of 28.5% among the craniotomy cohort, 26.1% among MMA embolization patients, and 20.3% among burr hole patients (Table 7). Rates of postoperative seizure were highest among craniotomy patients (16.9%, vs. 11.0% for MMA embolization and 9.2% for burr holes). Other complication rates were comparable between cohorts, albeit with craniotomy associated with slightly higher infection and PE rates, and MMA embolization associated with slightly higher rates of MI.

Patterns of postoperative cost at 90 days followed trends demonstrated at 30 days. The 90-day all-cause median postoperative cost for the MMA embolization cohort was $10,343. This cost profile was intermediate-approximately 50% higher than the burr hole cohort ($6,877) but 19% lower than the craniotomy cohort ($12,714; p < 0.001). This cost hierarchy was predominantly driven by inpatient utilization. Again, patients who underwent MMA embolization in addition to an open surgical procedure incurred a median 90-day postoperative cost of $24,496, with stroke or TIA rates of 40.4% within this combined cohort, substantially higher than that of any single-modality group (Table 9).

**Table 9:** The 90-day healthcare costs stratified by procedure group, including single-modality and combined procedure cohorts TIA: transient ischemic attack; MI: myocardial infarction; PE: pulmonary embolism; IQR: interquartile range; MMA: middle meningeal artery Categorical variables are reported as N (%). Continuous variables are reported as median (IQR). To maintain privacy, values where n < 10 have been redacted. Test statistic for categorical variables and continuous variables represent Chi-square values and F-statistics, respectively. Threshold for statistical significance was set at p < 0.05

	Combined	Burr holes only	Craniotomy only	MMA embo only	Test statistics	p-value
	(n = 188)	(n = 6021)	(n = 6822)	(n = 257)		
Number of days with outpatient encounter	14.0 (8.0-22.0)	10.0 (5.0-19.0)	12.0 (6.0-22.0)	11.0 (6.0-19.0)	37.0	<0.001
90-day postoperative outpatient costs ($)	6284.7 (2995.5-12148.9)	3623.0 (1369.1-9825.5)	4644.9 (1701.0-12201.1)	4212.5 (1745.5-8926.0)	36.5	<0.001
Number of days with inpatient encounter	8.0 (4.0-21.0)	3.0 (1.0-7.0)	6.0 (2.0-14.0)	2.0 (0.0-9.0)	312.6	<0.001
90-day postoperative inpatient costs ($)	24496.8 (4674.1-73868.6)	563.6 (76.0-5847.6)	2257.4 (341.4-20129.1)	891.4 (0.0-17217.8)	530.5	<0.001
90-day complication rates						
Seizure	28 (14.9%)	558 (9.3%)	1150 (16.9%)	28 (10.9%)	161.7	<0.001
Stroke or TIA	76 (40.4%)	1208 (20.1%)	1923 (28.2%)	68 (26.5%)	139.8	<0.001
MI	7 (3.7%)	79 (1.3%)	128 (1.9%)	<10 (<2%)	11.8	0.008
PE	<10 (<3%)	152 (2.5%)	253 (3.7%)	<10 (<2%)	16.7	<0.001
Infection	<10 (<3%)	63 (1.0%)	127 (1.9%)	<10 (<0.5%)	17.4	<0.001

## Discussion

This large-scale, claims-based analysis of 16,291 patients receiving treatment for nontraumatic SDH provides a broad, real-world portrait of the early clinical and economic footprint of MMA embolization for SDH across the United States. Our data confirm that MMA embolization is rapidly gaining traction as a primary therapeutic strategy for SDH, surging from a negligible proportion to approximately 30% of all treated cases by 2023. This exponential adoption since 2018 underscores the neurosurgical community’s use of MMA embolization as a minimally invasive alternative to traditional surgical approaches.

Geographic variation

The observed geographic variations in procedural selection for SDH highlight key differences in regional adoption patterns. The relative concentration of MMA embolization in the Northeast likely reflects its role as an early epicenter for clinical adoption, academic promotion, and specialized neurointerventional training. Conversely, the uniform distribution of burr hole and craniotomy procedures aligns with their long-standing status as the established standard of care, ensuring broad availability across diverse healthcare settings. In contrast, MMA embolization remains disproportionately centered within specialized neurointerventional hubs and high-volume tertiary care facilities.

Clinical outcomes and patient selection

A crucial finding is that the MMA embolization cohort presented with a significantly higher burden of baseline comorbidities, notably a nearly doubled rate of coagulopathy (22.2%). This suggests that, in practice, interventionalists are selecting MMA embolization for patients deemed high-risk for open surgery. Despite this clinical complexity, single-modality MMA embolization demonstrated a highly favorable safety profile with the lowest rate of in-hospital mortality (2%) and a discharge-to-home rate mirroring burr hole evacuation (67%) and exceeding craniotomy (50%).

Our findings regarding reintervention rates are consistent with and extend the evidence from recent RCTs. In the EMBOLISE trial, adjunctive MMA embolization reduced reoperation rates from 11.3% to 4.1% at 90 days [6]. In our real-world cohort, the overall rates of secondary open surgery (burr hole or craniotomy) following initial MMA embolization were 12.4% at 90 days, which is within the accepted recurrence range for traditional surgical methods alone and is particularly encouraging given the higher baseline comorbidity burden of this cohort. These findings compare favorably with the MAGIC-MT and STEM trials, which also reported significant reductions in recurrence with adjunctive embolization [4,9]. Our study extends the findings of Nia et al., who used the TriNetX Analytics Network to characterize early trends in MMA embolization [10], by providing detailed economic and health resource utilization data that were not previously available in the literature.

The postoperative stroke and TIA rates observed in our cohort warrant careful interpretation. At 30 days, stroke or TIA rates were 19.5% for MMA embolization, 22.7% for craniotomy, and 15.1% for burr holes, substantially higher than the rates reported in clinical trials. This discrepancy is likely multifactorial. In administrative claims data, stroke and TIA codes may erroneously capture both new ischemic or hemorrhagic events and pre-existing chronic conditions (including the SDH itself) or incidental diagnoses that appear in discharge billing. Furthermore, our patient population had significantly higher baseline rates of cardiovascular risk factors, coagulopathy, prior stroke, and atrial fibrillation compared to curated clinical trial populations. These inflated administrative stroke rates are an important limitation and should be interpreted with caution; nevertheless, the observation that combined MMA embolization plus open surgery was associated with the highest stroke or TIA rate (41.2%) warrants prospective investigation.

Economic Implications and Combination Therapy

Our findings demonstrate that burr hole evacuation was the lowest cost procedure at 90 days ($6,877), craniotomy was the highest ($12,714), and single-modality MMA embolization was intermediate ($10,343). Index hospitalization cost for embolization was highest, in part due to multi-step interventional billing, encompassing arterial access, selective carotid catheterization, and diagnostic angiography in addition to the embolization itself. These costs must also be interpreted in the context of greater comorbidity burden in the embolization cohort.

At the extreme high end of the healthcare cost spectrum are the high resource utilization and risk associated with combined open surgical and embolization treatment. Patients who underwent both an open procedure and MMA embolization had a median 90-day postoperative cost of $24,496 and a 90-day stroke or TIA rate of 40.4%, findings which, to our knowledge, have not been previously reported in the literature. Interpreting these results is challenging in the context of the available MarketScan data, but this cohort may include patients who were planned for staged treatment or patients who failed initial monotherapy and required crossover treatment, groups that may both be at inherently higher clinical risk. Together, these findings strongly suggest that combining modalities carries substantial clinical and economic risk and warrants focused study to understand patient selection and complication mitigation in this group.

Limitations

Despite the strengths of utilizing a large national claims database to provide high-volume, real-world data, this study is subject to several key limitations inherent to its design. First, the retrospective nature and reliance on administrative data (ICD and CPT codes) preclude the ability to capture crucial clinical variables that influence outcomes, such as the initial patient presentation (e.g., Glasgow Coma Scale score), the presence of mass effect, or the degree of neurological improvement postprocedure. Second, claims data provide no radiographic information, meaning we cannot assess the size, mass effect, or morphology of the SDH, the completeness of the MMA embolization, or the degree of radiographic resolution, which are central to judging treatment efficacy. Results must be interpreted in the context of these factors inherent to retrospective administrative study design, which may result in residual confounding and selection bias from nonrandomized treatment assignment.

Third, it is important to acknowledge that there is no ICD code specifically designating chronic SDH; the nontraumatic SDH designation (ICD-9 432.1; ICD-10 I62.0x) is used to capture chronic and subacute presentations, as well as some spontaneous acute bleeds, which may introduce a degree of diagnostic heterogeneity into our cohort. This coding limitation is inherent to all administrative database studies of cSDH and limits the ability to restrict the analysis exclusively to chronic presentations. Fourth, the MarketScan dataset captures commercial and Medicare supplemental patients, introducing a potential selection bias and limiting the generalizability of the cost data to uninsured or purely Medicare populations. The relatively lower number of MMA embolization patients compared to burr hole and craniotomy cohorts may also affect results; however, this is unavoidable as our goal was to analyze the early adoption period, and large-scale databases take several years to publish claims data. Finally, while the focus on 30- and 90-day outcomes maximized the comparability of the cohorts given the recent adoption of MMA embolization, the relatively short follow-up duration prevents definitive conclusions regarding long-term recurrence rates beyond the subacute phase, a critical factor for cSDH management. Longer-term prospective studies are critical.

## Conclusions

This study provides compelling evidence from a large claims database that MMA embolization has rapidly expanded as a key treatment modality for chronic SDH. In real-world practice, it is utilized in a high-risk patient population, where it is associated with favorable short-term clinical outcomes and an intermediate cost profile relative to traditional surgical approaches. While the adoption trend is clear and single-modality observations are encouraging, the exceptionally poor outcomes linked with combined surgical and embolization procedures highlight a crucial target for quality improvement and protocol refinement. Collectively, these real-world data demonstrate the growing utilization of MMA embolization in contemporary neurosurgical practice and underscore the need for high-quality, long-term prospective trials to establish its precise efficacy and definitive role in the cSDH treatment algorithm.
